# Use of Antidepressants among Patients Diagnosed with Depression: A Scoping Review

**DOI:** 10.1155/2021/6699028

**Published:** 2021-03-15

**Authors:** Nirmal Raj Marasine, Sabina Sankhi, Rajendra Lamichhane, Nabin Raj Marasini, Nim Bahadur Dangi

**Affiliations:** ^1^Western Health Science Academy, Pokhara, Kaski, Nepal; ^2^Pharmaceutical Sciences Program, School of Health and Allied Sciences, Pokhara University, Kaski, Nepal; ^3^Department of Public Health, Asian College for Advance Studies, Lalitpur, Nepal; ^4^Department of Public Health, La Grande International College, Kaski, Nepal

## Abstract

**Introduction:**

Depression is a major global health problem with a relatively high lifetime prevalence and significant disability. Antidepressants are the most effective medications used for the treatment of depression. Hence, this study is aimed at summarizing the studies on antidepressant use among patients diagnosed with depression.

**Method:**

PubMed, Embase, Web of Science, Scopus, and Google Scholar were searched for literature (2000-2019) using keywords such as depression, drug utilization, antidepressants, prescription, serotonin reuptake inhibitor, serotonin and norepinephrine reuptake inhibitor, tricyclic antidepressants, and atypical antidepressants.

**Results:**

Antidepressant users were mostly females, married people, housewives, lower-income people, employees, and highly educated people, as they were found to be more prone to develop depression than their counterparts. Selective serotonin reuptake inhibitors (SSRIs), such as sertraline, were most commonly prescribed among depressive patients.

**Conclusion:**

Our study suggested that out of five major antidepressant drugs available for the treatment of depression, selective serotonin reuptake inhibitors are preferred over others because of their better side effects and tolerability profile.

## 1. Introduction

Depression is a common mental disorder and a major cause of functional disability [1, 2]. According to the World Health Organization (WHO), by 2020, it will be the second-highest known cause of worldwide disability [3, 4]. Depression is characterized by a sad mood, pessimistic thought, lowered interest in day-to-day activities, poor concentration, insomnia or increased sleep, significant weight loss or gain, decreased energy, continuous feelings of guilt and worthlessness, decreased libido, and suicidal thoughts occurring for at least two weeks [5, 6]. Depressed patients can be of any gender, age, or background. Due to fear of stigmatization associated with mental disorders, patients lack to seek medical treatment in their early stages [7–9]. To maintain normal human health in patients, drugs play a crucial role. Antidepressant drugs are the most widely used and are most effective in the treatment of depression [10, 11]. For many years, tricyclic antidepressants (TCAs) have been the drug of choice for treating depression in patients [12–14]. Many new antidepressants with better tolerance and broader indications have been discovered because of an increase in the prevalence of depression throughout the world [15]. This results in the gradual replacement of conventional drugs such as TCAs and monoamine oxidase inhibitors (MAOIs) by selective serotonin reuptake inhibitors (SSRIs), serotonin-norepinephrine reuptake inhibitors (SNRIs), and atypical antidepressants [7, 16, 17]. The most appropriate antidepressants should be selected according to symptoms and patient characteristics, with adequate dose and duration of therapy, to enhance the treatment success rate [18, 19]. However, other factors, such as adverse effect profiles, cost, safety profile, history of prior medication treatment, and patient preference, are important in the initial selection of antidepressants and should be considered by physicians [20, 21]. The optimal use of antidepressants could reduce individual distress, along with the social burden of depression [22]. The aim of the analysis of prescribing patterns is to evaluate the prescription habits of medical practitioners and to suggest necessary modifications if required to make drug therapy rational and cost-effective [6, 16]. The use of antidepressants has increased within the last two decades [23, 24]. As a result of the increased prescription and use of antidepressants, the need for information regarding the actual prescribing practices has become vital to maintain patient safety as well as to ensure that the optimal therapeutic outcome is achieved, especially when the nature of the side effects of these drugs is considered. Hence, this study is aimed at summarizing the studies on antidepressant use among patients diagnosed with depression.

## 2. Methods

### 2.1. Search Strategy

Databases such as PubMed, Embase, Web of Science, Scopus, and Google Scholar were searched for studies published between January 2000 and December 2019. The main Medical Subject Heading (MeSH) terms used for literature search within the databases were depression, drug utilization, antidepressants, and prescription. Other keywords used were serotonin reuptake inhibitor, serotonin and norepinephrine reuptake inhibitor, tricyclic antidepressants, and atypical antidepressants. For the second round of search, the keyword “antidepressant” was combined with “drug utilization”, “prescription”, “depression”, “depressive patients”, “systematic reviews”, and “narrative reviews”. The bibliographies of relevant articles were also searched for more studies that were not identified in the original database search. All the abstracts and studies were screened for their relevance and discarded those that did not fit our selection criteria. Only those studies that were identified as potentially relevant to our study title were retrieved and fully reviewed.

### 2.2. Study Selection

#### 2.2.1. Inclusion Criteria

Inclusion criteria are the following: (1) literatures of varying methodologies such as observational, cross-sectional, and retrospective studies, survey, and case reports, (2) studies conducted on all patients aged ≥18 years with a primary diagnosis of depression and prescription of at least one antidepressant drug, (3) studies that mainly focused on antidepressant utilization or antidepressant prescription pattern among patients with depression, and (4) full-text articles, published in peer-reviewed journals, in years lying between 2000 and 2019 and available in English language.

#### 2.2.2. Exclusion Criteria

Exclusion criteria are the following: (1) reviews, clinical trials, descriptive studies, pilot studies, editorials, case series, conference abstracts, letters, commentaries, posters, qualitative interviews, and book chapters; (2) studies conducted on patients younger than 18 years with no depression and not prescribed with antidepressants; pregnant or lactating mothers; those with a history of psychotic, bipolar disorder, or drug abuse; and those with cognitive impairment; (3) studies that did not focus on antidepressant utilization or antidepressant prescription pattern among patients with depression; and (4) studies published in non-peer-reviewed journals before 2000 and after 2019 and available in languages other than English.

#### 2.2.3. Study Outcomes

Our study outcomes were the demographic factors and types of antidepressants used among patients with depression.

## 3. Results

**Figure 1:**
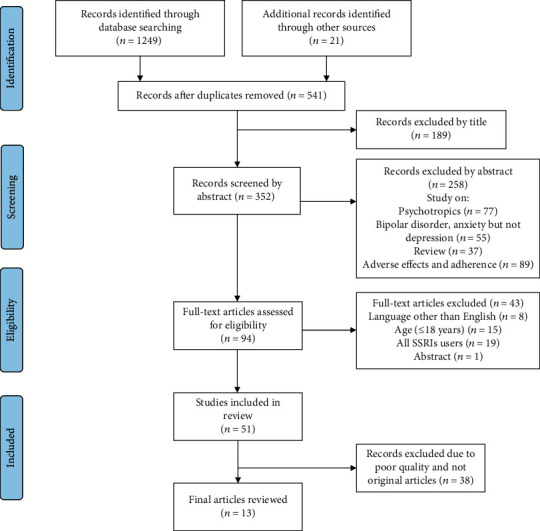
Data screening and extraction.

The literature search process is summarized in Figure 1. A total of 51 studies were included in our review; only 13 original articles were reviewed. The studies were conducted in India [25], Bangladesh [26], Malaysia [27], Nigeria [28], Singapore [29], China [30], Italy [31], Saudi Arabia [32], USA [33], Australia [34], Germany [35], Canada [36], and Netherlands [37]. The summarized main findings of the studies are presented as follows (Table 1).

## 4. Discussion

We identified thirteen studies through this review, and the majority of the patients were in the economically productive age group of 40-50 years [25–30, 32–37]. Conversely, the findings of a study from Italy showed that the mean age of the patients receiving antidepressant prescriptions was more than 50 years [31]. Our review showed that the majority of the patients receiving antidepressants for the treatment of their depression were females [26, 28–41]. This could be due to hormones that are associated with the regulation of the menstruation cycle and pregnancy affecting mood in females. These alterations in hormonal regulation cause dysregulation of the stress response, which makes them more sensitive to depression and often shows magnified neuroendocrine responses to even low levels of stress [28, 38, 39, 42]. Women play multiple roles in family and society, such as homemakers, spouses, mothers, professionals, and caregivers. These multiple responsibilities may be the source of increased stress that might have led to depression in them [43, 44]. In many societies, until today women are not given equal respect, they are considered less powerful with low status, they cannot make a choice, and they are sexually abused, which all results in the development of depression in them [5, 42]. In contrast, a study from our neighboring country, India, depicted more depressive males than females, which could be due to more stress at work, a monotonous lifestyle with no entertainment, low income, and economic burden of family [25]. Evidence suggests that depression is associated with various psychological factors, such as loneliness, lack of family care and affection, poor family support, insufficient time with children, high use of emotional coping, low level of spirituality, stressful incidents, poor health, and dependency [25, 38]. Sedentary lifestyle, lack of physical exercise, lack of hobby, irregular dietary habits, smoking, and taking alcoholic beverages or substance abuse are also interconnected with depression [25, 30, 35]. Continuous arguments, stressful daily routines, unsupportive spouses, continuous discouragement, lack of family time or husbands or wives going to other countries for employment, and ignorance from family members may be the reason for more married, housewives, and lower-income people being vulnerable to depression [25, 26, 28, 29, 31, 35, 36, 45]. One study showed that a spouse's weekly working hours are greatly associated with the partner's risk of developing depression and suicidal thoughts [46]. This means that long working hours not only affect individuals' own mental health but also affect their spouses [46]. Unsatisfactory job, lower income, high level of physical activity, time pressure, lack of encouragement, promotion, and job security are associated with lowering self-esteem and hence could be the reason for taking antidepressants by a high number of employees involved in paid works [25, 29, 35]. Similarly, being concerned about more profit or suffering a continuous loss in business may also lead to depression in people involved in self-employed business [28]. Our review showed that education is another source of depression in many people. People with a higher education background becomes the victim of depression when they do not get a job equivalent to their qualification. On the other hand, in the job they got involved, they have to work as instructed with unsatisfactory payment and no opportunity to implement their knowledge and skills due to which they feel lack of challenges in their work along with lack of intellectual growth in them [25, 29, 31, 36]. However, other studies have displayed less educated people as victims of depression [26, 35]. These people work as machine operators, laborers, farmers, and unskilled manual workers, where there is more physical and psychological-related stress along with less respect from other employees.

The prescribing pattern of antidepressants for patients with depression varies across different countries. This could be due to differences in availability and antidepressant prices as well as variations in recommendations in each country's national guidelines [28, 36]. Medical treatment of depression not only improves the mental health of patients but also increases their physical and social performance, making them optimistic and encouraged towards life [32]. Our review revealed that SSRIs are the dominant antidepressants prescribed over TCAs, SNRIs, and other atypical antidepressants for the treatment of depression [25–27, 29–37]. The preference of psychiatrists for SSRI prescription over other antidepressants could be because of the advantages they offer to the patients. Antidepressants other than SSRIs nonselectively inhibit the reuptake of norepinephrine, dopamine, and serotonin into presynaptic vesicles and affect adrenergic, cholinergic, postsynaptic serotonin, and histaminic receptors in the brain, which are unrelated to depression, leading to intolerable adverse effects [47]. SSRIs do not cause life-threatening adverse effects, such as overdose-related cardiotoxicity and CNS toxicity, as they do not show receptor antagonism [48]. Additionally, they can be administered once daily, require less dose titration than TCAs, are safer, and show fewer side effects compared to other antidepressants [26, 31, 33–37, 49]. Hence, it could be safer and effective for many patients. In contrast, a study showed TCAs as the most commonly prescribed antidepressants despite SSRIs being more advantageous [28]. This could be due to the affordability and easy availability of TCAs over SSRIs. In developing countries, the affordability of drugs plays an important role in the continuation of treatment because many low-income families cannot afford expensive medicines. The majority of the population has to rely on government insurance policies to obtain drugs for their treatment. Many people buy their prescribed antidepressants from the government hospital as they are available at cheaper prices than in retail pharmacies. Such regional differences along with cultural differences and country economy also create huge differences in the prescription of antidepressants [50, 51]. Our review showed that sertraline was the most frequently prescribed SSRI, followed by others such as escitalopram, fluoxetine, paroxetine, and fluvoxamine. Amitriptyline was commonly prescribed among TCAs, venlafaxine and duloxetine among SNRIs, and mirtazapine and bupropion among atypical antidepressants.

### 4.1. Limitation and Strength of the Study

There are certain limitations to our study. Literature published in languages other than English was excluded, which might be associated with language bias. The data used were observational, cross-sectional, retrospective, survey, and case reports only. This does not provide direct insight into the changing trends of prescribing behaviors of physicians over time in patients and may reflect a bias. Likewise, based on clinical setting and physician variables, prescription pattern varies. This study does not provide information on such variables and clinical appropriateness of antidepressants used. However, systematic search strategy and review of types of studies included which are of about two decades are the strength of this study. Additionally, a number of characteristics associated with antidepressant prescription such as age, gender, education, marital state, socioeconomic status, and all other sociodemographic factors are identified. Hence, the findings of this study are expected to have a good impact on the education of psychopharmacology.

## 5. Conclusion

Our study revealed that the majority of antidepressant users were aged between 40 and 50 years, females, married, housewives, lower income, and highly educated people. SSRIs were found to be highly prescribed over TCAs, SNRIs, MAOIs, and atypical antidepressants. Among the prescribed SSRIs, sertraline was the dominant SSRI. The result of this study suggests the further need for high-quality studies, which may consider the use of data sources like clinical files and patient self-reports, and also includes reports on whether antidepressants were prescribed to treat physical or mental symptoms.

## Figures and Tables

**Table 1:** Study, sample, methods, and major findings of the studies.

Study	Objective	Methodological review	Major findings
Tejashwini et al. [25],	To evaluate the antidepressant use pattern and associated adverse drug reactions (ADR).	Prospective observational study *n* = 598 Age: ≥18 years (April 2016 to September 2016)	Major victims of depression: married, employees, and housewives with secondary education. Antidepressant receivers (majority): males (57.86%) and people aged between 41and 60 years. Commonly prescribed antidepressants: fluoxetine (50.27%), followed by sertraline (40.29%), amitriptyline (25.31%), and escitalopram (3.43%).
Islam et al. [26],	To evaluate the antidepressant prescription pattern following WHO prescribing indicators in two teaching hospitals.	Hospital-based descriptive cross-sectional study. *n* = 300 Age: 18-60 years (June 2015 to June 2016)	Antidepressant receivers (majority): people aged between 18 and 27 years, married women, housewives, less educated, unemployed, and the lower-income group from a rural area. Commonly prescribed antidepressants: SSRI (sertraline followed by escitalopram, citalopram, and fluoxetine), TCA (amitriptyline followed by imipramine), SNRI (venlafaxine), and atypical group (mirtazapine).
Nahas and Sulaiman [27],	To evaluate the antidepressant prescription pattern among depressive men in Malaysia.	Cross-sectional study *n* = 107 Age: ≥18 years (from May 2015 to ten-month period)	Mean age: 49.9 years. Commonly prescribed antidepressants: SSRIs (72.9%), followed by TCAs (10.3%), SNRI (8.4%), and MAOIs (2.8%).
Kehinde et al. [28],	To evaluate the antidepressant utilization pattern in the tertiary care hospital in Lagos.	Retrospective study *n* = 683 Age: ≥18 years (January 2013 to December 2014)	Major victims of depression: people aged between 31 and 45 years, females (67.2%), married (57%), and self-employers (49.7%). Commonly prescribed antidepressants: TCAs (61.3%) and SSRIs (38.7%). Most frequently prescribed: amitriptyline (60.6%) and sertraline (20.2%).
Soh et al. [29],	To evaluate the antidepressant prescription pattern in a psychiatric department of a general hospital in Singapore.	Retrospective study *n* = 206 Age: ≥18 years (January 2013 to December 2013)	Mean age: 50 years The majority of the patients were females (63.6%), married (70.9%), highly educated (46.1% had tertiary education), and full-time employees (60.2%). Commonly prescribed antidepressants: SSRIs (75.5%), followed by atypical antidepressants (13.5%) and TCAs (8.5%).
Chen et al. [30],	To evaluate the prevalence and prescription of antidepressants used in depression in Asia.	Cross-sectional study *n* = 956 Age: ≥18 years	Mean age: 45.2 years Antidepressant receivers (majority): females Commonly prescribed antidepressants: sertraline (19.6%), escitalopram (18.6%), and mirtazapine (16.1%).
Trifirò et al. [31],	To evaluate the antidepressant prescription pattern in Italian primary care.	A prospective, observational cohort study *n* = 1,377 Age: ≥18 years (1 January 2007 to 1 June 2008)	Mean age: 52 years Antidepressant receivers (majority): 45-64 years age group, where most of them were females (71.5%), homemakers (31.1%), married or cohabiting (60.4%), highly educated (44.2%), and current or former smokers and alcohol consumers and obese (53.5%). Frequently prescribed antidepressants: SSRIs (paroxetine 25.9% and escitalopram 18.4%), SNRIs (venlafaxine 11.6% and duloxetine 5.6%) (80.2%), and TCA (2%).
Alhulwah et al. [32],	To evaluate the long-time users of antidepressants at Riyadh Military Hospital.	Cross-sectional study *n* = 120 Age: ≥18 years (July 2009 to September 2010)	Mean age: 42 years Antidepressant receivers (majority): females (57.5%) and 35-50 years age group (46%) Commonly prescribed antidepressants: SSRI (61.7%), atypical antidepressant (14.2%), and TCA (11.7%), respectively.
Prukkanone et al., [33]	To evaluate the antidepressant prescription pattern in the hospice program.	Retrospective cohort study *n* = 17 Age: ≥18 years (June 2007 and December 2008)	Most users were female. Commonly prescribed antidepressants: SSRIs (prescribed in 9 out of 10 patients).
Shiroma et al. [34],	To measure adherence and determine the antidepressant prescription pattern in patients with major depression.	Retrospective study, *n* = 1,058 Age: ≥18 years (patient treated between August 2005 and September 2008)	Average age: 46 years Antidepressant receivers (majority): females (64%) and 15-86 years age group Commonly prescribed antidepressants: fluoxetine (in two-thirds of patients), TCAs, and other SSRIs.
Bauer et al. [35],	To evaluate the recent antidepressant prescription pattern in European countries.	Prospective, observational study *n* = 3,468 Age: ≥18 years (May 2004 to September 2005)	Mean age: 46.8 years Antidepressant receivers (majority): females (68.2%), married, less educated, unemployed, paid workers, and smokers. Commonly prescribed antidepressants: SSRIs (63.3%), followed by TCAs (26.5%) and SNRIs (13.6%).
Beck et al. [36],	To evaluate the antidepressant utilization in relation to sociodemographic variables, in Canada.	Cross-sectional survey *n* = 2,145 Age: ≥18 years (May-December 2002)	The majority of antidepressant users were age group (25-64 years), females, married, lowest-income group, and people with higher education.
Meijer et al. [37],	To evaluate the prescribing pattern in patients using new antidepressants.	Observational cohort study *n* = 1,251 Age: ≥18 years (1995-1997)	Median age: 41 years Female: 64.1% Commonly prescribed antidepressants: sertraline (52.7%), paroxetine (31.2%), fluoxetine (9.2%), and fluvoxamine (7%).

## Data Availability

The raw data used to support the findings of this study are made available from the corresponding author upon reasonable request.
